# UniStatus - a cross-sectional study on the contamination of uniforms in the Danish ambulance service

**DOI:** 10.1186/s13104-015-1057-4

**Published:** 2015-03-25

**Authors:** Heidi Storm Vikke, Matthias Giebner

**Affiliations:** Medical department, Falck Denmark A/S, 6000 Kolding, Denmark

**Keywords:** Prehospital, Uniform, Hygiene, Bacterial contamination, Infection control

## Abstract

**Background:**

Patients are at risk of contracting infections due to the presence of disease-causing microorganisms that can be transmitted from the medical staff’s uniforms to the patient. The dual purpose of this study was to examine the contamination level of the uniforms worn by ambulance staff after a shift and to test the effect of washing of the uniform with and without a detergent containing acetic peroxide.

**Methods:**

This was a cross-sectional study in which 30 ambulance staff uniforms were randomly selected for inclusion and divided into two groups. Before washing, 90 prints were performed with specific agar plates to determine bacterial contamination and to establish the prevalence of a variety of microorganisms. Group A uniforms were washed with a detergent without acetic peroxide; Group B uniforms were washed with a detergent containing acetic peroxide.

**Results:**

Before washing, the 90 prints had an average colony-forming units (CFU) of potentially pathogenic bacteria of 68.89 per 25 cm^2^ and a prevalence of: *E. coli* and *Pseudomonas* 0%, *Bacillus cereus* 27.78% (CI 95% ± 9.80), *Clostridium* and *Enterococcus* 2.22% (CI 95% ± 1.96), *Staphylococcus aureus* 21.11% (CI 95% ± 7.80). After washing, CFU was reduced to 3.09 (CI 95% ± 5.04) per 25 cm^2^ in Group A and to 1.47 (CI 95% ± 4.77) per 25 cm^2^ in Group B. The prevalence of specific bacteria in either group was 0%, except for *S. aureus* which had a prevalence rate of 4.40% (CI 95% ± 6.10) in Group A. The difference between the contamination degrees of the two groups was not significant in either test (p > 0.05).

**Conclusion:**

Potentially pathogenic bacteria are detectable on ambulance staff uniforms when a shift ends. Optimal prevention of bacterial infection may be achieved by daily changing, washing at a minimum of 60 degrees Celsius and use of a detergent containing acetic peroxide.

## Background

National and international prehospital staff have an interest in obtaining thorough knowledge about the contamination level of prehospital uniforms and, thus, about the risk of infection to which patients are exposed in the prehospital setting. Several international studies have examined various aspects related to health staff uniforms including, among others, the presence of methicillin-resistant *Staphylococcus aureus* (MRSA) [1,2], the importance of the washing procedure for microbiological reduction [3] and the hygiene culture among health professionals [4]. These studies, however, have all concerned hospital or home care staff.

Studies have demonstrated that uniforms in healthcare services become contaminated with microorganisms, among others through the staff’s contact with patients [4]. Thus, a clinical study demonstrated the spreading of infection from the uniform to the patient and the transmission of infection from patient to uniform and then to another patient [5]. Several other studies have also shown that there is a risk of transmitting bacteria by direct contact [6]. This indicates that uniforms in prehospital healthcare services may also constitute a source of infection and thus a health risk for patients.

In the Region of Southern Denmark, more than 100,000 patients are transported by ambulances annually (quote by: Niels Thorup, Regional Director, Falck Denmark A/S, Region of Southern Denmark, 2014). The staff in these ambulances carries standardized uniforms consisting of a jacket and a pair of trousers that are washed in accordance with instructions based on an accredited standard. Presumably, the washing procedure reduces the number and types of microorganisms on clothes after washing [3]. However, our literature search revealed no evidence of the exact significance of washing on daily practice in Danish prehospital settings.

Furthermore, several studies show that hospital uniforms can be contaminated with microorganisms [1,7,8]. The most commonly occurring microorganisms are *S. aureus* and *Enterococcus*, among several others [1,4]. Still, our literature search produced no studies examining which microorganisms are involved in the bacteriological contamination of staff uniforms in Danish ambulance services.

We therefore examined the extent to which the ambulance staff uniforms are contaminated with potentially pathogenic microorganisms *before* washing. Specifically, we examined the potentially pathogenic bacterial contamination and the prevalence rate for *E. coli*, *B. cereus*, *Clostridium*, *S. aureus, Enterococcus* and *Pseudomonas*. We also examined to which extent the bacterial contamination was reduced through use of a washing detergent with and without acetic peroxide. All uniforms were washed at 60 degrees Celsius and tumble-dried for 1 hour.

## Methods

A cross-sectional study was carried out at a random number of selected ambulance stations in the Region of Southern and Central Denmark. In accordance with standard procedures, uniforms were washed locally in the stations where the staff is employed. Practical sampling was applied, and the collected uniforms had all been used in the ambulance service within the past 24 hours. The collection took place over a period of 7 days. The staff was not *blinded* as they are usually responsible for the washing of uniforms.

A total of 30 ambulance staff uniforms were selected randomly after they had been worn during a shift lasting a minimum of 8 hours. Before washing, all 30 uniforms were contact-printed with specific 25 cm^2^ agar plates or inoculated using swab sticks for bacterial mapping.

In order to determine the level of potentially pathogenic contamination, blood agar plates were used, and the samples were incubated at 37 ± 1 degrees Celsius for 24 ± 3 hours. To determine the prevalence of *B. cereus*, blood agar plates were used and subsequently incubated at 30 ± 1 degrees Celsius for 24 hours. *S. aureus* prevalence was determined using rabbit plasma fibrinogen (RPF) agar plates which were incubated at 37 ± 1 degrees Celsius for 18-24 hours and, if necessary + 24 hours. For the examination of *Enterococcus* prevalence, we used Slanetz agar and an incubation temperature of 44 ± 1 degrees Celsius for 48 ± 4 hours. The *Clostridium* prevalence was examined with tryptose sulphite cycloserine (TSC) at an incubation temperature of 37 ± 1 degrees Celsius for 24 ± 3 hours. The *Pseudomonas* prevalence was examined with rapid Pseudomonas agar at an incubation temperature of 36 degrees Celsius for 22-33 hours. Finally, the *E. coli* prevalence was examined by Tryptone Bile X-glucornide (TBX) agar at an incubation temperature of 44 ± 1 degrees Celsius for 21 ± 3 hours. Table 1 presents the laboratory methods used.

**Table 1:** **Laboratory methods**

**Bacterial focus**	**Agar plate/swab stick**	**Incubation time/temperature**
*Potentially pathogenic bacteria*	Blood agar	37 ± 1 degrees Celsius for 24 ± 3 hours
*B. cereus*	Blood agar	30 ± 1 degrees Celsius for 24 hours
*S. aureus*	RPF agar	37 ± 1 degrees Celsius for 18-24 hours*
*Enterococcus*	Slanetz agar	44 ± 1 degrees Celsius for 48 ± 4 hours
*Clostridium*	TSC	37 ± 1 degrees Celsius for 24 ± 3 hours
*Pseudomonas*	Rapid *Pseudomonas* agar	36 degrees Celsius for 22-33 hours
*E-coli*	TBX agar	44 ± 1 degrees Celsius for 21 ± 3 hours

*If necessary, an additional 24 hours.

Prints were obtained from the torso, the thighs and the wrist area which previous studies have shown are the most contaminated locations on healthcare uniforms [4,9].

### Random selection of the intervention groups

The uniforms were randomly selected for washing and drying in two different groups before prints were taken with agar plates. After the prints were taken, the uniforms in Group A were washed in a domestic washing machine at 60 degrees Celsius with the Tex-Power 350 detergent that does not contain acetic peroxide. The uniforms were then dried in a domestic tumble dryer at medium heat for 60 minutes. The uniforms in Group B were also washed in a domestic washing machine at 60 degrees Celsius, but with the Tex Des 60 detergent which *does* contain acetic peroxide. The uniforms were then dried at medium heat in a domestic tumble dryer for 60 minutes. After washing and drying, specific agar prints were taken again from all uniforms, precisely as before the washing.

All agar prints were collected locally in the participating ambulance stations. The samples were then stored at < 5 degrees Celsius during transportation to the laboratory. Incubation and laboratory analysis were performed in accordance with a DANAK-accredited method (quote by: Marianne Sloth Johansen, AnalyTech A/S, Denmark, 2014). The laboratory specified the results as number of CFU per 25 cm^2^.

### Statistical analysis

Descriptive statistics were performed. The overall level of potentially pathogenic contamination before and after washing is given by mean, standard deviation (SD), minimum and maximum values and reported with a 95% confidence interval (CI). To test the difference in reduction of mean CFU, *Student’s t-test* was performed with an alpha = 0.05. The prevalence of each specific bacterium before and after washing was presented with a number of positive prints associated with a percentage with CI 95%. The chi-squared test was used to test for significant difference (alpha = 0.05) between the prevalence of the chosen bacteria in the two groups. Furthermore, proportion tests were performed to test for significant differences in the percentage reduction among and between the groups. Our data were not 100% normally distributed; we therefore tested our results of the *Student’s t-test* with a non-parametric test which showed similarity.

### Ethical considerations

The study is exempted from ethical approval since it neither involved humans nor animals. To ensure that the study complied with normal standards and instructions, all uniforms were washed after they had been tested, i.e. before they were used in emergency medical services again.

## Results

### Potentially pathogenic contamination level

The potentially pathogenic contamination per uniform print before washing in Group A and Group B varied from 0 to 200 CFU per 25 cm^2^. A total of 90 uniform prints were contaminated with an average of 68.89 CFU per 25 cm^2^ (SD ± 65.83).

### Potentially pathogenic contamination level before and after washing

Before washing, the average potentially pathogenic contamination level for the 45 selected uniform prints in Group A was 82.49 CFU per 25 cm^2^ (SD ± 65.21). For the prints in Group B, the average potentially pathogenic contamination level was 55.29 (SD ± 64.30). After washing, the contamination level was reduced to an average of 3.09 (SD ± 5.04) CFU per 25 cm^2^ for prints in Group A. In Group B, the average CFU was reduced to 1.47 (SD ± 4.77) per 25 cm^2^. As presented in Table 2, there was no significant difference in the average contamination degree between the two groups *before* washing (p > 0.05), but there was a significant difference between the two groups *after* washing (p < 0.05).

**Table 2:** **Degree of general potentially pathogenic contamination**

**CFU per 25 cm2**	**Group A**	**Group B**	p-value*
*n*	*45*	*45*	
Mean	82.49	55.29	0.06
***Before washing***			
SD	65.21	64.30	
Minimum	0.00	0.00	
Maximum	200.00	200.00	
CI 95%	19.59	19.32	
**After washing**			
Mean	3.09	1.47	0.02
SD	5.04	4.77	
Minimum	0.00	0.00	
Maximum	29.00	30.00	
CI 95%	1.52	1.43	

*Student’s t-test.

### Reduction in potentially pathogenic bacteria after washing

As presented in Table 3, comparison of the average CFU per 25 cm^2^ in the groups before and after washing showed a reduction of general, potentially pathogenic bacteria of 96.25% for the 45 prints in Group A where the uniforms were washed without a disinfectant detergent. Moreover, an average reduction of 97.35% was found for the 45 prints in Group B where the uniforms were washed using a disinfectant detergent. The difference between the CFU reductions in the groups came close to significance (p = 0.06).

**Table 3:** **Reduction in general potentially pathogenic contamination after washing**

**CFU per 25 cm** ^**2**^	**n**	**Before washing**	**After washing**	**Reduction (%)**	p-value*
**Group A**	45	82.49	3.09	79.40 (96.25)	*0.06*
**Group B**	45	55.29	1.47	53.83 (97.35)	

*Proportion tests.

### General prevalence

As demonstrated in Table 4, *Pseudomonas* and *E. coli* were not found on any of the prints in the study. Out of 90 prints, we found *S. aureus* on 21.11% (CI 95% ± 7.80), *B. cereus* on 27.78% (CI 95% ± 9.80) and E*nterococcus* as well as *Clostridium* on 2.22% (CI 95% ± 1.96).

**Table 4:** **Prevalence before washing all uniforms (** ***n = 90*** **)**

	**Number**	**%**	**SE**	**CI 95%**
***S. aureus***	19	21.11	0.04	± 7.80
***B. cereus***	25	27.78	0.05	± 9.80
***Enterococcus***	2	2.22	0.01	± 1.96
***Clostridium***	2	2.22	0.01	± 1.96

### Prevalence before and after washing

As presented in Table 5, the prevalence rates of the specific microorganisms were distributed differently after random selection. *S. aureus* was verified on 24.40% of the prints in Group A and on only 17.80% of the prints in Group B (p > 0.05). The prevalence of *B. cereus* was 15.60% in Group A and 40% in Group B. This difference between the two groups was significant (p < 0.05). *Enterococcus* was verified on 4.40% of uniform prints in Group A and on 0% of the prints in Group B *Clostridium* was not found on any of the prints in Group A, but the prevalence of this bacterium was 4.40% on the 45 uniform prints in Group B.

**Table 5:** **Prevalence of specific bacteria before washing, grouped (n = 45)**

	**Group A**			***Group B***			
	**Number**	**%**	**CI 95%**	**Number**	**%**	**CI 95%**	P-values*
***S. aureus***	11.0	24.4	±12.5	8.0	17.8	±11.2	*0.44*
***B. cereus***	7.0	15.6	±10.6	18.0	40.0	±14.3	*0.01*
***Enterococcus***	2.0	4.4	±6.1	0.0	-	-	-
***Clostridium***	0.0	-	-	2.0	4.4	±6.1	-

*Chi-squared tests.

As shown in Table 6, only two prints were positive for *S. aureus* among the 45 prints in Group A after washing, which equals a prevalence of 4.4% (CI 95% ± 6.1) for uniforms washed without disinfecting detergent. In Group B, no prints tested positive to *S. aureus*. None of the 90 prints divided into Group A and Group B tested positive to *Pseudomonas, E. coli, B. cereus, Enterococcus* or *Clostridium* after washing (p was not calculated due to non-prevalence).

**Table 6:** **Prevalence of specific bacteria after washing, grouped (n = 45)**

	**Group A**			***Group B***			
	**Number**	**%**	**CI 95%**	**Number**	**%**	**CI 95%**	
***S. aureus***	2.0	4.4	± 6.1	0.0	-	-	

## Discussion

Several healthcare related studies have found that healthcare-related uniforms were contaminated with various potentially pathogenic bacteria and thus associated with a risk of patient infection [5,10,11]. However, no Danish study has previously examined the bacterial contamination of prehospital uniforms which leaves us with no results for comparison. However, since our results indicate that an average print from a prehospital uniform has similar or fewer potentially pathogenic bacterial colonies than found on uniforms from in-hospital studies [12], we suggest that the contamination level of uniforms in prehospital settings is, to some extent, comparable to the contamination level of in- hospital uniforms.

Our findings of potentially pathogenic bacteria correspond to previous findings in hospital-based studies [1,12]. Our results indicate that *S. aureus*, E*nterococcus* and *Clostridium* are present on used prehospital uniforms with a presumed bacterial susceptibility comparable to that of patient isolates; however, we did not collect patients’ samples and therefore cannot compare the results to those of patient isolates. Nevertheless, the present bacteria are already known to cause healthcare associated infections (HAIs).

The fact that we found a lower prevalence of E*nterococcus* than in a previous study focusing on nurse uniforms [4] is probably explained by the fact that nurses’ tasks are not identical to those performed by staff in the prehospital setting. For example, prehospital staff may perform much fewer tasks related to personal care, e.g. hygiene following a restroom visit, etc., which are known to be associated with infection. Although we did not find *E. coli* or *Pseudomonas*, we cannot dismiss that prehospital uniforms could be contaminated with either of these microorganisms. Nevertheless, we assume that the contamination level and hence the risk of infecting patients is, indeed, limited.

Washing and tumble-drying is known to aid bacterial reduction, especially as prehospital uniforms dictate a maximum washing temperature of 60C. However, our results show that washing *and* tumble-drying in domestic machines for 1 hour, regardless of the choice of washing powder, did not eliminate all microorganisms. The washing machine, the tumble-dryer and the environment could all have caused this contamination, and it would have been beneficial if we had collected environmental samples which would have provides us with knowledge of a possible correlation between contamination of uniforms and contamination of machines and/or the environment. In addition, we could also have tested the temperature in the tumble dryer and the washing machine.

Unfortunately, our methodology has a number of limitations that must be addressed. Firstly, the results of the washing process are compromised given that the two groups compared did not have the same degree of contamination at baseline. However, we were unable to ensure this as this was an *in situ* study*.* Secondly, due to practicalities such as timeframe and financial limitations, we did not perform a sample size calculation, which could have ensured a sample size sufficiently large to obtain a significant difference. Therefore, we acknowledge that this study should be considered a pilot study, and that a larger future study requires further consideration.

There is also a risk of bias associated with the position of the prints before and after washing. Although we tried to ensure that prints were taken from the same location before and after washing, there is a margin of error associated with the outcome as we cannot guarantee that the prints were placed in the same location before and after washing. Another important issue that must be emphasized is the variation in our results caused by the use of domestic washing machines; however, we did intend to illustrate the result during the practical laundering and handling process in the prehospital setting. We used three uniform sampling locations which have previously been shown to be the most contaminated locations for assessing the mean degree of contamination of the uniforms, and we assessed the mean degree of contamination. The sampling technique itself was well known by the environmental laboratory performing the analysis, and its feasibility for that purpose is well-established in current literature. It was therefore decided not to further test its reproducibility.

As mentioned, the number of international studies on the effect of different washing methods is very limited [7,10], and no previous studies in the Danish ambulance services can substantiate our findings on bacterial contamination or on the quality of the used washing methods. At the same time, evidence of the risk associated with the transmission of microorganisms between staff and patients is very limited. Furthermore, no Danish correlation studies between bacterial CFU and incidence of infection in the prehospital setting have been performed. Nor have any studies sought to correlate different methods of washing with the incidence of infections. In Denmark, no comprehensive registration of HAIs is presently in place and therefore such analysis is not possible. We recommend that such registration be introduced to enable studies on the association between contamination degree and risk of infection.

Overall, further studies are needed on prehospital bacterial contamination and hygiene interventions. Increased evidence of contamination degrees, their underlying causes and the effect of hygiene interventions and procedures will, all things being equal, strengthen infection control in the prehospital setting.

## Conclusion

The results of this study indicate the presence of a contamination level which - in several respects - underpins the need to comply with existing hygiene instructions in the prehospital setting. Daily change of uniform, washing at a minimum of 60 degrees Celsius and use of a disinfectant detergent (acetic peroxide) reduce the degree of bacterial contamination and thus reduce the risk that patients become infected. The results concerning a potentially pathogenic contamination level and the prevalence of specific microorganisms, including *B. cereus, Clostridium, S. aureus* and *Enterococcus*, stress the need for daily change of uniform and a high hygiene standard in daily prehospital work with patients to avoid increasing the risk that microorganisms are transmitted from ambulance uniforms to patients.
